# Structure of the intact Tom20 receptor in the human translocase of the outer membrane complex

**DOI:** 10.1093/pnasnexus/pgae269

**Published:** 2024-07-26

**Authors:** Jiayue Su, Xuyang Tian, Ziyi Wang, Jiawen Yang, Shan Sun, Sen-Fang Sui

**Affiliations:** State Key Laboratory of Membrane Biology, Beijing Frontier Research Center for Biological Structure, Beijing Advanced Innovation Center for Structural Biology, School of Life Sciences, Tsinghua University, Beijing 100084, China; State Key Laboratory of Membrane Biology, Beijing Frontier Research Center for Biological Structure, Beijing Advanced Innovation Center for Structural Biology, School of Life Sciences, Tsinghua University, Beijing 100084, China; State Key Laboratory of Membrane Biology, Beijing Frontier Research Center for Biological Structure, Beijing Advanced Innovation Center for Structural Biology, School of Life Sciences, Tsinghua University, Beijing 100084, China; School of Life Sciences, Cryo-EM Center, Southern University of Science and Technology, Shenzhen 518055, Guangdong, China; State Key Laboratory of Membrane Biology, Beijing Frontier Research Center for Biological Structure, Beijing Advanced Innovation Center for Structural Biology, School of Life Sciences, Tsinghua University, Beijing 100084, China; State Key Laboratory of Membrane Biology, Beijing Frontier Research Center for Biological Structure, Beijing Advanced Innovation Center for Structural Biology, School of Life Sciences, Tsinghua University, Beijing 100084, China; School of Life Sciences, Cryo-EM Center, Southern University of Science and Technology, Shenzhen 518055, Guangdong, China

**Keywords:** translocase of the outer membrane complex, Tom20, mitochondria, cryo-electron microscopy

## Abstract

The translocase of the outer membrane (TOM) complex serves as the main gate for preproteins entering mitochondria and thus plays a pivotal role in sustaining mitochondrial stability. Precursor proteins, featuring amino-terminal targeting signals (presequences) or internal targeting signals, are recognized by the TOM complex receptors Tom20, Tom22, and Tom70, and then translocated into mitochondria through Tom40. By using chemical cross-linking to stabilize Tom20 in the TOM complex, this study unveils the structure of the human TOM holo complex, encompassing the intact Tom20 component, at a resolution of approximately 6 Å by cryo-electron microscopy. Our structure shows the TOM holo complex containing only one Tom20 subunit, which is located right at the center of the complex and stabilized by extensive interactions with Tom22, Tom40, and Tom6. Based on the structure, we proposed a possible translocation mode of TOM complex, by which different receptors could work simultaneously to ensure that the preproteins recognized by them are all efficiently translocated into the mitochondria.

**Significance statement**

For the maintenance of mitochondrial stability, precursor proteins necessitate transportation from the cytoplasm to mitochondria via the TOM complex. Recognition of these precursor proteins occurs through the TOM complex receptors Tom20, Tom22, and Tom70, followed by their entry into mitochondria through Tom40. Despite the crucial role of Tom20 in this process, its precise location within the TOM complex has remained elusive. In this study, we captured the structure of the human TOM holo complex containing intact Tom20. The obtained structure suggests the TOM holo complex does not fully conform to the C2 symmetry and there is only one Tom20 binding. Based on this structure, we present an insight into the composition of the whole TOM complex and its transport mechanism.

## Introduction

Mitochondria, as double-membraned organelles ubiquitous in eukaryotic cells, provide energy to living organisms (1). This important organelle comprises the outer membrane, inner membrane (IM), intermembrane space (IMS), and matrix (1). Among them, the IM can be further divided into the inner boundary membrane (IBM), the crista membrane, crista junctions, and mitochondrial contact sites (2–5). In order to maintain mitochondrial stability, thousands of proteins are required to work together, and most of them are encoded by nuclear genes and synthesized in cytosol (6–8). The translocase of the outer mitochondrial membrane (TOM) complex plays a major role in transporting these proteins into mitochondria (1, 9).

The TOM complex comprises seven distinct subunits: Tom40, Tom20, Tom22, Tom70, Tom5, Tom6, and Tom7 (9, 10). Tom40, forming the TOM complex channel, displays a β-barrel structure which is surrounded by three α-helical subunits Tom5, Tom6, Tom7 (11–20). Tom6 and Tom7 seem to have opposite effects for the TOM complex (7, 21, 22). Tom6 contributes to TOM complex stability by interacting with Tom22, but Tom7 reduces its stability. Another three subunits, Tom20, Tom22, and Tom70, serve as receptors recognizing precursor proteins (23). Most preproteins have short signal sequence in the N-terminal that can be recognized by receptor of TOM complex and then transported into mitochondria (4).

Recent evidences indicate that the impair of mitochondria is related to many human diseases, especially the damages of the receptor proteins of the TOM complex (24–28). Tom20 and Tom22 are associated with Parkinson's disease pathogenesis by affecting mitochondrial protein import (29, 30). Meanwhile, Tom20 is related to age-related hearing loss (31); Tom22 correlates with hepatocyte development and regeneration, which may be responsible for liver mitochondrial disease (32). Additionally, Tom70 is associated with human cardiac diseases and the severe acute respiratory syndrome coronavirus 2 (SARS-CoV-2) (33, 34).

So far, the structures of many TOM complexes from fungi and human have been resolved utilizing cryo-electron microscopy (cryo-EM) (35–38). The highest resolutions of the TOM complex from fungi and human have been resolved to 3.06 Å (36) and 2.53 Å (39), respectively. However, most of these TOM complexes are the core complex including Tom22, Tom40, Tom5, Tom6, and Tom7. Only two low-resolution structures of TOM complexes including part of Tom20 have been reported recently (39, 40). The inadequate information of localizations and structures of receptor proteins Tom20 and Tom70 hinders the understanding of the mechanism of preprotein entering mitochondria.

Here, we determined the structure of the human TOM holo complex containing the entire Tom20 by chemical cross-linking of the mitochondria. This structure is at a resolution of about 6 Å, and exhibits a dimeric conformation without C2 symmetry. Only one Tom20 subunit is observed in this structure. Based on the structure, we propose a possible mode for Tom20, Tom22, and Tom70 to participate in the preprotein translocation.

## Result

### Cross-linking of mitochondria

In order to obtain the structure of the complete TOM complex, we used chemical cross-linking to stabilize the association among components. Because TOM is a transmembrane protein complex, we used disuccinimidyl glutarate (DSG), a water-insoluble and membrane-permeable cross-linker, to crosslink the purified mitochondria prior to protein isolation. Subsequently, digitonin (DIG) was used to extract membrane protein and the TOM complex was obtained by FLAG-tag affinity resin and gel filtration chromatography as previously reported (37, 39) (Fig. S1).

### Overall structure of the human TOM holo complex containing the intact Tom20

**Fig. 1.**
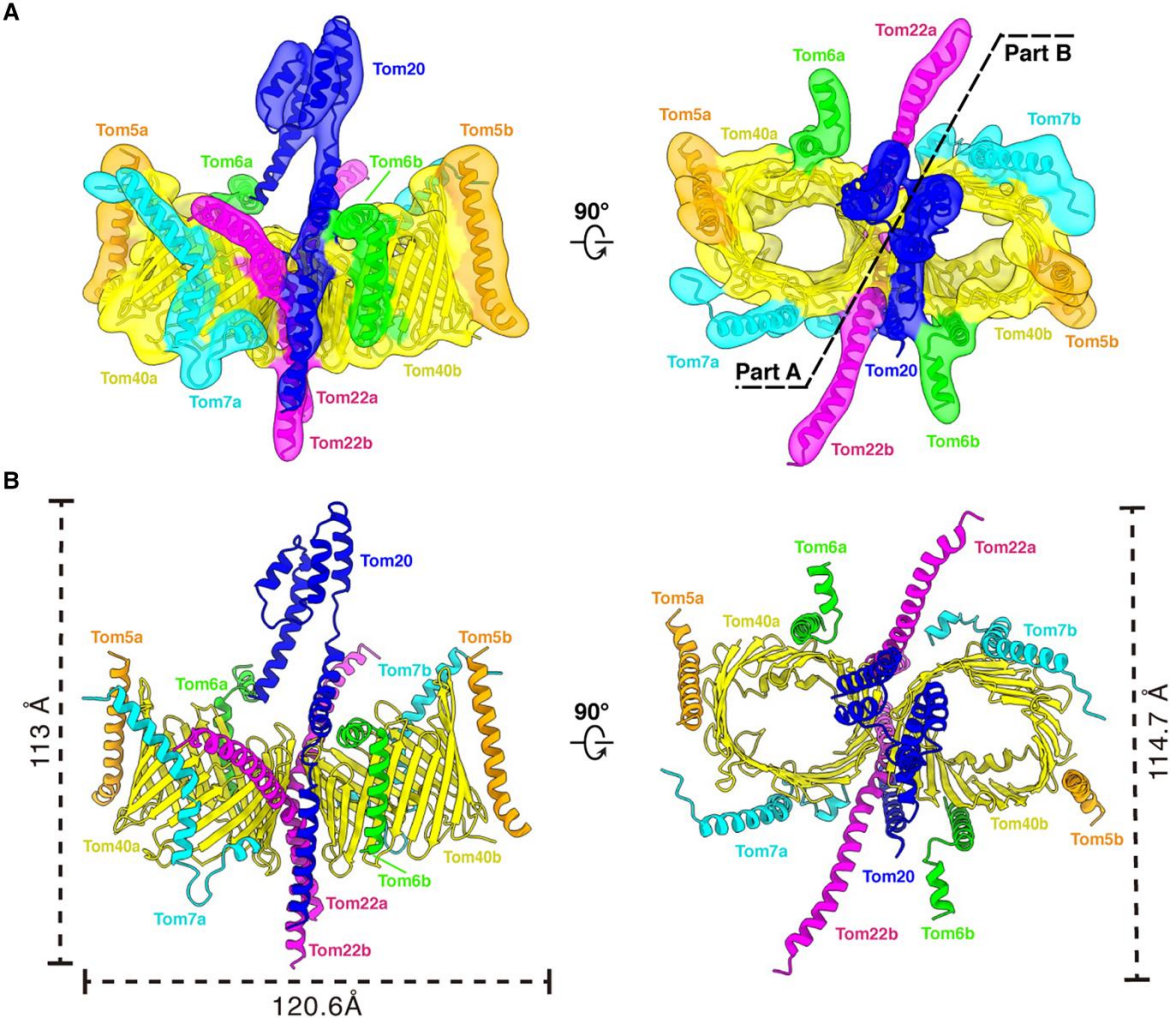
Overall structure of the human TOM holo complex including whole Tom20 at 6 Å. (A) Cryo-EM density map overlapped with the atomic model of the human TOM holo complex containing whole Tom20 in two different views. (B) Cartoon representation of the atomic model.

The structure of the isolated human TOM complex (hTOM) is resolved at an overall resolution of 6.87 Å without imposing any symmetry. By applying local masks during refinement, the resolutions for the core region and the Tom20 reached 5.62 Å and 4.72 Å, respectively (Fig. S1 and S2, Table S1). Similar to the previously determined cryo-EM structures (39), the hTOM core complex is a binocular-like dimer with each monomer (monomer a and monomer b) consisting of Tom40, Tom22, Tom5, Tom6, and Tom7 (Fig. 1). Tom40, serving as the pivotal core subunit of the TOM complex, forms the channel for preprotein translocation (41–44) and is surrounded by Tom22, Tom5, Tom6, and Tom7. The dimension of the dimeric hTOM complex is 113 Å by 120.6 Å by 114.7 Å (Fig. 1).

**Fig. 2.**
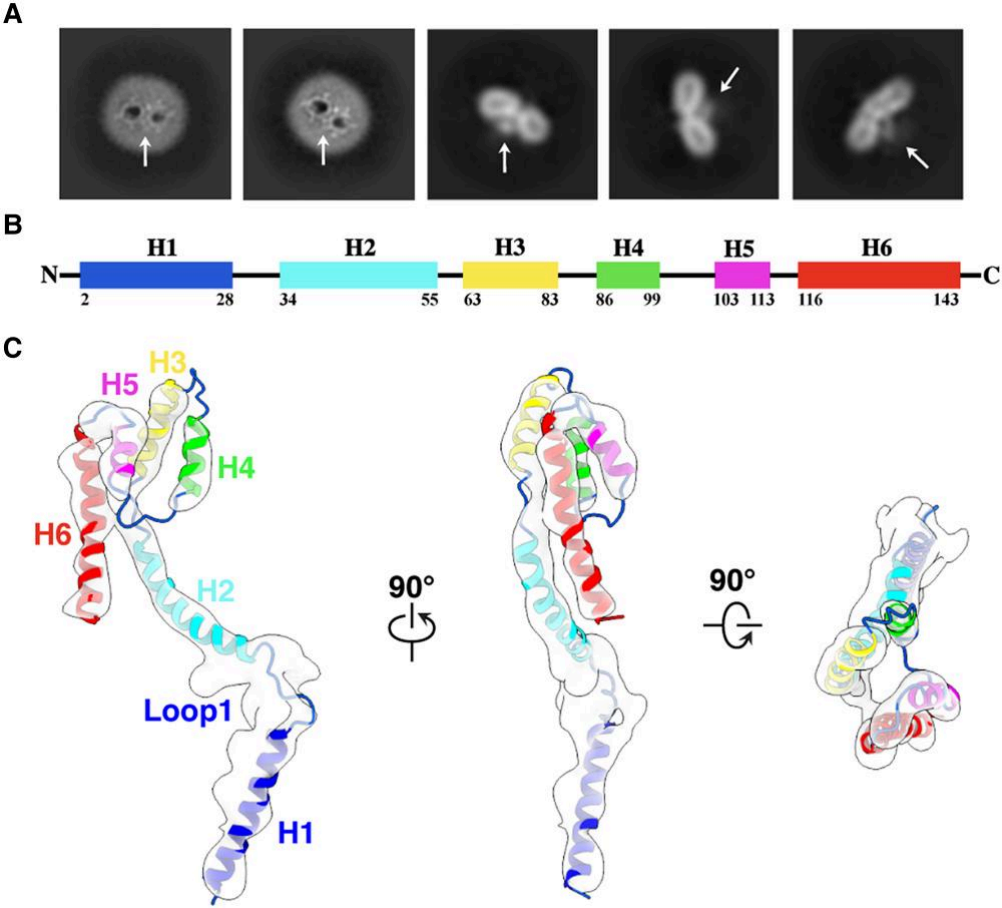
The position and structure of Tom20. (A) 2D classification of the TOM complex purified from the cross-linked mitochondria. White arrows indicate the position of Tom20 subunit. (B) Schematic of the secondary structural elements of Tom20. (C) Tom20 (cartoon representation) and the EM density map (transparent white) shown in three different views.

Surprisingly, except the densities corresponding to the core complex, we observe extra densities both in the images of two-dimensional class averages and the reconstructed density map (Figs. 1A and 2A). A transmembrane α-helix passes through the micelle from the IMS to the cytosolic side, bends at the membrane surface, and continues to extend upward above the interface of the two pores, forming a hook-like structure at the end (Figs. 1A and 2B). The density of the hook shows very clearly that it is made up of four α-helices. The whole density contains six α-helices, which matches the secondary structure prediction of Tom20 by PSIPRED showing that Tom20 is composed of six α-helices (Fig. 2B, S3A). To build the model of Tom20, the AlphaFold predicted model was rigid-body fitted into the map first. Three α-helices, H1 (V2-R28), H2 (F34-S55), and H6 (P116-D143), are matched well with the density (Fig. S3B). We then modified the positions of α-helices H3 (A63-A83), H4 (Y86-V99), and H5 (P103-T113) according to the map to generate the most possible model of the final Tom20 structure (please refer to the Materials and methods for details) (Fig. 2C, S3C-G). This Tom20 structure exhibits a different conformation compared to our previous study (39). Possible explanations for this difference are: the cross-linking was applied to the mitochondria in this work instead of the purified TOM complex in the previous work, and only C1 symmetry was applied in data processing in this work.

**Fig. 3.**
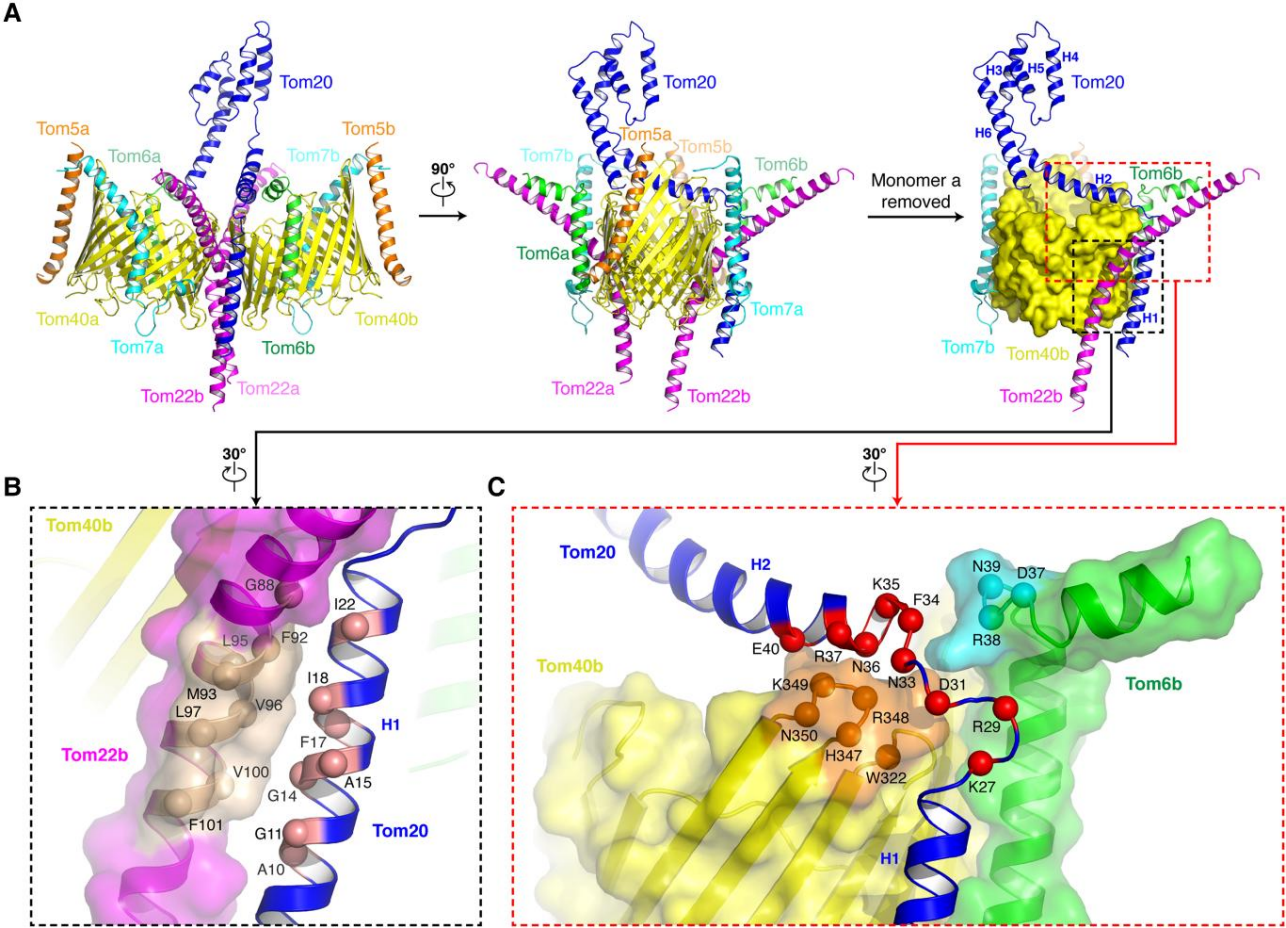
Interactions of Tom20 and the hTOM core complex. (A) The different views of the hTOM holo complex to show the position of Tom20. (B) Interaction between Tom20 and Tom22b. (C) The interaction of Tom20 with Tom40b and Tom6b.

Although the hTOM core complex shows two-fold symmetry, there is only one Tom20 in the holo complex. In general, Tom20 is located right at the center of the hTOM holo complex. The N-terminal transmembrane α-helix H1 consists mainly of hydrophobic amino acids and is situated outside of Tom40b and in contact with nearby Tom22b (Fig. 1 and 2C and 3A). The subsequent α-helix H2 protrudes from the membrane and lies above the edge of Tom40b. It is almost perpendicular to H1 and lifts in the distal part. The loop (Loop1) linking H1 and H2 forms a kink and helps H2 bend relative to H1 (Fig. 1 and 2C and 3A). The cytosolic domain composed of α-helices H3-H6 forms an antiparallel α-helix bundle and faces monomer a (Fig. 2C). The C-terminal of Tom20 extends above the pore of Tom40a.

### Interactions between Tom20 and the hTOM core complex

We are able to analyze how the receptor Tom20 binds to the hTOM core complex based on the structure of the hTOM holo complex (Fig. 3). Two contact areas are observed and they are within the membrane and at the membrane surface, respectively (Fig. 3A).

The first interface is between the transmembrane α-helices of Tom20 and Tom22b in the membrane (Fig. 3B). As expected, this interface is enriched by hydrophobic residues from both Tom20 (A10, G11, G14, A15, F17, I18, and I22) and Tom22 (G88, F92, M93, L95, V96, L97, V100, and F101), suggesting that strong hydrophobic interactions between Tom20 and Tom22 may stabilize the transmembrane region of Tom20 to facilitate the binding of Tom20 to the hTOM core complex.

Followed the transmembrane α-helix of Tom20 is a long loop connecting to the α-helix H2. As shown in Fig. 3B, this loop together with the N-terminal region of H2 is clamped steady by two claws extended from Tom40b and Tom6b, respectively (Fig. 3C). This contact area is largely composed of polar residues including eight residues from Tom20, four residues from Tom40b and three residues from Tom6b, indicating that electrostatic or hydrogen-bonding interactions dominate the interactions. Additionally, two pairs of positively charged residue and aromatic residue, K27 from Tom20 and W322 from Tom40b, and R38 from Tom6b and F34 from Tom20, provide two potential cation–π interactions. These extensive interactions between Tom20 with Tom40b and Tom6b stabilize the almost vertically bent conformation of H2 with respect to H1 (Fig. 3C).

It should be noticed that Tom20 in the hTOM holo complex is different from Tom20 in the NcTOM holo complex (40) both in position and conformation (Fig. S4), which may be due to the different species, sample preparation and image processing. In detail, the transmembrane helix of NcTom20 is far away from that of NcTom22, and NcTom20 interacts with the N-terminus of NcTom22 at the cytosolic membrane surface by electrostatic interactions (40). In contrast, the transmembrane helices of hTom20 and hTom22 are tightly held together in the membrane by hydrophobic interactions (Fig. 3).

### Conformational changes of the hTOM core complex upon Tom20 binding

**Fig. 4.**
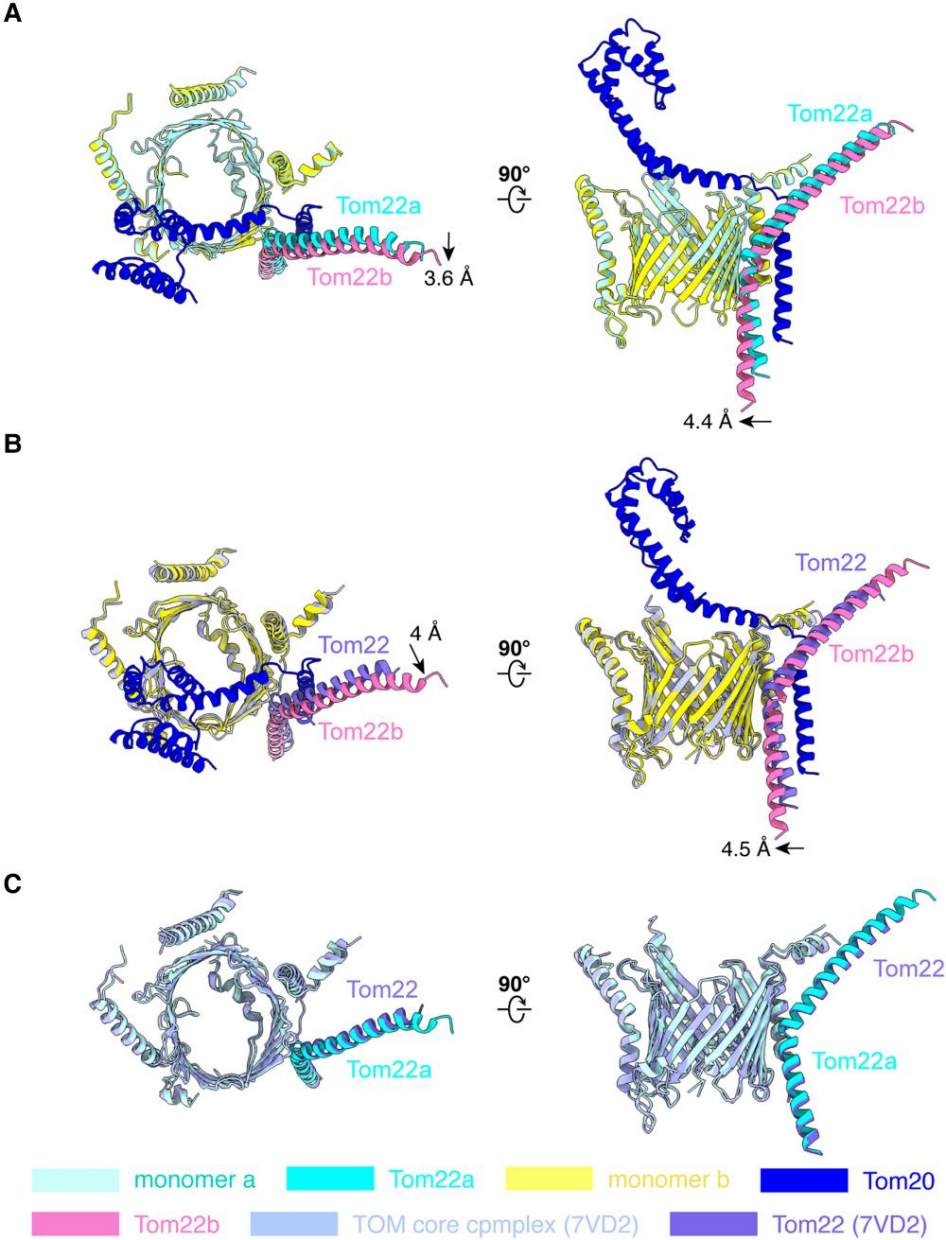
Conformational changes in the hTOM complex containing Tom20. (A) Structural comparison between monomer a and monomer b of the hTOM complex containing Tom20. (B) Structural comparison between monomer b of hTOM complex containing Tom20 and the monomer of hTOM complex without Tom20 (PDB: 7VD2). (C) Structural comparison between monomer a of hTOM complex containing Tom20 and the monomer of hTOM complex without Tom20 (PDB: 7VD2).

As described above, Tom20 interacts with only one Tom22, Tom22b, although the hTOM core complex contains two copies of Tom22. Thus, it is reasonable to infer that the two Tom22 subunits may have different conformations due to the asymmetric binding of Tom20. Indeed, structural comparison between monomer a and monomer b of the hTOM holo complex shows obvious difference in Tom22, while other subunits are conformationally similar (Fig. 4A). To adapt the binding of Tom20 into the hTOM core complex, of the two Tom22 subunits, the one (Tom22b) that interacts with Tom20 undergoes a displacement. Compared to the C-terminal of Tom22a, the C-terminal of Tom22b moves towards the center of the core complex by about 4.4 Å (Fig. 4A). The N-terminal of Tom22b is pushed away from Tom20 for about 3.6 Å in comparison with Tom22a (Fig. 4A). These movements of Tom22b provide sufficient space for the accommodation of Tom20. In contrast to Tom22, the interaction between Tom20 and Tom6 results in a slight conformational change in Tom6b compared to Tom6a (Fig. S5).

Moreover, when the structure of the hTOM holo complex obtained in this work is compared to that of the hTOM complex without Tom20 (PDB: 7VD2) from previous study (39), obvious conformational change exists only in the Tom22 subunit contacting with Tom20 (Fig. 4B, C, S6) (39). The structure of 7VD2 is a symmetric dimer composed of two monomers, each of which contains Tom40, Tom22, Tom5, Tom6, and Tom7. In comparison with Tom22 from 7VD2, the C-terminal of Tom22b moves towards the center of the complex by about 4.5 Å, while the N-terminal moves about 4 Å in the direction away from Tom20 (Fig. 4B), which is very similar to the difference between Tom22b and Tom22a in the hTOM holo complex. Consistent with this observation, the conformation of Tom22 from 7VD2 is very similar to that of Tom22a in the hTOM holo complex (Fig. 4C). All of these structural analyses support the conclusion that Tom20 binding to the hTOM core complex could lead to the conformational change of Tom22 that interacts with Tom20.

### Possible preprotein transportation mode by TOM complex

**Fig. 5.**
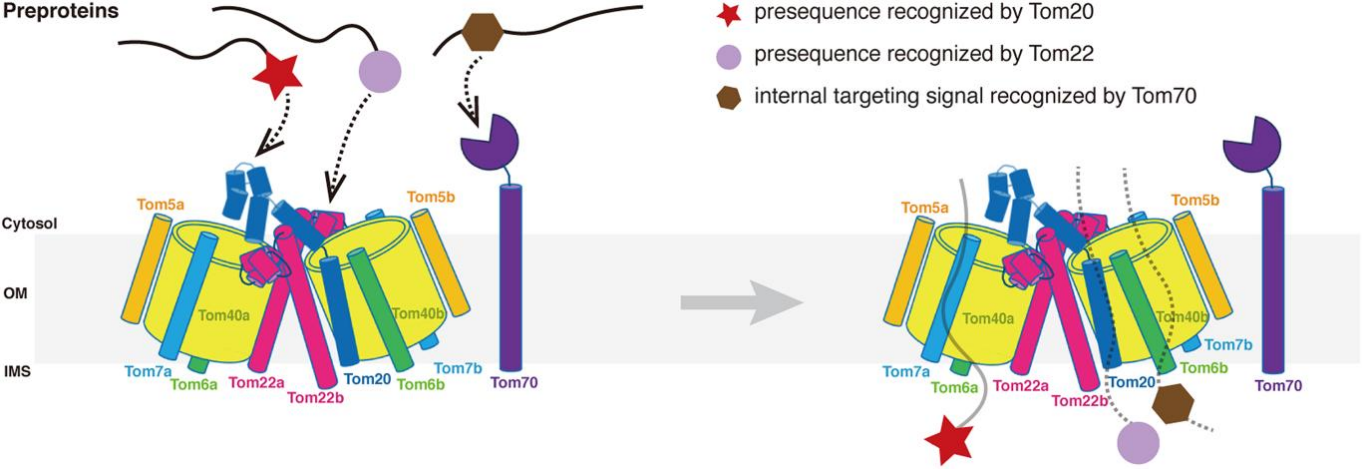
Possible preprotein transportation mode by TOM complex. The preprotein recognized by Tom20 could enter one of the two pores formed by Tom40. Meanwhile, the other pore could transport preproteins recognized by other receptors, such as Tom22 or Tom70.

This work presents the structure of the hTOM holo complex containing the complete Tom20 receptor, offering valuable insights into how this complex assembles and functions to transport precursor proteins into mitochondria. While the subunits of the hTOM core complex, Tom40, Tom22, Tom5, Tom6, and Tom7, have stoichiometry of 1:1:1:1:1 (28), it does not mean that the structure of the hTOM holo complex conforms to C2 symmetry. Our structure clearly shows that the hTOM holo complex is a two-hole structure without C2 symmetry and contains only one Tom20 subunit. Thus, the preprotein recognized by Tom20 could only enter one of the two pores formed by Tom40, possibly Tom40a as it is close to the cytosolic domain of Tom20 (Fig. 5). Meanwhile, the other pore could transport preproteins recognized by other receptors, such as Tom22 or Tom70 (Fig. 5). Taking together, our results suggest a possible translocation mode of the TOM complex, by which different receptors could work simultaneously to ensure that the preproteins recognized by them are all efficiently translocated into the mitochondria (Fig. 5).

## Materials and methods

### TOM complex purification

The TOM complex purification was performed the same as previously described (38) with additional step of mitochondria cross-linking. After isolation of mitochondria, the pure mitochondria were resuspended with buffer B (20 mM MOPS pH 7.4, 150 mM KCI, 10% (v/v) glycerol). 5 mM DSG (Thermo Scientific, 20593) was added to the solution and incubated for 1 hour at 4°C for the mitochondria cross-linking. Then the cross-linking reaction was quenched by 50 mM Tris–HCl (pH 8.0). The cross-linked mitochondria were added to 1% (v/v) digitonin (BIOSYNTH, number: D3200) for extraction. The following purification of TOM complex was the same as previously described (38).

### Cryo-EM sample preparation and data collection

In total, 4 μl of sample was applied to glow-discharged grid (R 0.6/1 Au, 400 mesh, Quantifoil). Grids were bolted by FEI Mark IV Vitrobot for 3.5 s and plunged to liquid ethane. Images were collected on a Titan Krios G3i TEM (Thermo Fisher Scientific) operated at 300 kV with a Gatan K3 Summit direct electron detector and GIF Quantum imaging energy filter. Two datasets were acquired with AutoEMation (written by Jianlin Lei) (45). The dataset was collected at a nominal magnification of ×81,000. The pixel size was 1.08 Å/pixel with defocus between −1.3 μm and −1.8 μm. The total dose on the detector was about 50 e/Å^2^ with the total exposure time of 2.56 s. Each micrograph stack contains 32 frames. Each micrograph was corrected for subregion motion correction and dose weighted using UCSF MotionCor2 (46).

### Single-particle image processing

Two thousand eight hundred and ninety-nine micrographs were collected and imported into cryoSPARC (47) for Patch CTF estimation (Mulit). Two hundred particles were manually picked by manual picker and applied to 2D classification. Good classes were selected and used as templates to run template picker. In total, 1,151,405 particles were picked with the particle diameter of 220 Å. Particles were extracted with bin4 and applied to 2D classification. After 2D classification, 329,764 good particles were kept to do ab-initio reconstruction with C1 symmetry. The best class accounted for 62.5% of total particles was selected and further subjected to 2D classification. Particles with low signal-to-noise ratio were discarded in this step. The remaining particles, encompassing both Tom20-containing and Tom20-lacking TOM complexes, were reextracted by bin1 and underwent a second round of ab-initio reconstruction with four classes. This step successfully separated the TOM complexes based on the presence or absence of Tom20. Here, 99,493 particles belonging to the TOM complex with Tom20 were subjected to refinement with C1 symmetry, which resulted in a 7.57 Å reconstruction. Further 3D classification of these particles with three classes revealed no significant conformational diversity. Thus, all these particles were subsequently refined with an overall mask, yielding a 6.87 Å reconstruction. By applying local masks during refinement, the resolutions for the core region and the Tom20 reached 5.62 Å and 4.72 Å, respectively. The lower resolution of the core region compared to Tom20 may be attributed to its environment. Detergent micelles surrounding the core could dampen the signal, leading to a lower signal-to-noise ratio and hindering high-resolution reconstruction. Otherwise, it also suggested potential heterogeneity within the core. The local masks were created based on the 6.87 Å map using Chimera (48). All resolutions were estimated in cryoSPARC by gold-standard Fourier shell correlation with a criterion of 0.143. Local resolutions were determined by cryoSPARC (Fig. S2).

### Model building

The structures of Tom40, Tom22, Tom5, Tom6, and Tom7 subunits were derived from the hTOM structure (PDB: 7VDD), which was first rigid-body fitted into the map using UCSF Chimera. All subunits fit well with the density map, except for Tom22b, which shows a minor displacement. Then, we manually adjusted Tom22b in COOT (49). To build the model of Tom20, the AlphaFold predicted model was first rigid-body fitted into the map. Three α-helices, H1 (V2-R28), H2 (F34-S55), and H6 (P116-D143), showed good agreement with the density map (Fig. S3B). In contrast, α-helices H3 (A63-A83), H4 (Y86-V99), and H5 (P103-T113) exhibited deviations from the map (Fig. S3B). We then manually placed them into the map in COOT. Based on the continuity of the densities, we first determined the position of H3. We then placed H4 and H5 into each of the two-remaining rod-like densities thus obtaining two models (Fig. S3C, D). We also rigid-fitted the model of the cytoplasmic domain of the rat Tom20 (PDB: 3AX3) into the map to obtain the third model (Fig. S3E), and modified the positions of helices according to the map to generate the fourth model (Fig. S3F). Among these models, the one with the highest correlation coefficient to the map is chosen as the final Tom20 structure (Fig. 2C, S3G). Subsequently, the individual models of each subunit were merged to generate the complete TOM holo complex model. Due to the resolution limitation, no real space refinement was further performed.

## Supplementary Material

pgae269_Supplementary_Data

## Data Availability

The atomic coordinate and the EM density map of the hTOM including the intact Tom20 (PDB: 8XVA, EMD-38694) have been deposited in the Protein Data Bank (www.rcsb.org) and the Electron Microscopy Data Bank (www.ebi.ac.uk/pdbe/emdb/). The local EM density maps of the core region (EMD-60277) and the Tom20 (EMD-60278) have been deposited in the Electron Microscopy Data Bank.
