# Supplementary material for: Structure of the intact Tom20 receptor in the human translocase of the outer membrane complex
Source: PNAS Nexus. 2024 Jul 26;3(7):pgae269. doi: 10.1093/pnasnexus/pgae269 (PMC11273160; doi:10.1093/pnasnexus/pgae269)
Supplement: pgae269_Supplementary_Data [file pgae269_supplementary_data.pdf]

Supplementary Information for

Structure of the intact Tom20 receptor in the human TOM complex

Jiayue Su[1]*, Xuyang Tian[1]*, Ziyi Wang[1], Jiawen Yang[2], Shan Sun[1]† and Sen-Fang Sui[1,2]†

[1] State Key Laboratory of Membrane Biology, Beijing Frontier Research Center for Biological Structure, Beijing Advanced Innovation Center for Structural Biology, School of Life Sciences, Tsinghua University, Beijing 100084, China.

[2] School of Life Sciences, Cryo-EM Center, Southern University of Science and Technology, Shenzhen 518055, Guangdong, China.

*These authors contributed equally to this work.

†Correspondence to: shansun@mail.tsinghua.edu.cn (S.S.), suisf@mail.tsinghua.edu.cn (S.-F.S.)

This PDF file includes:

Figures S1 to S6
Table S1

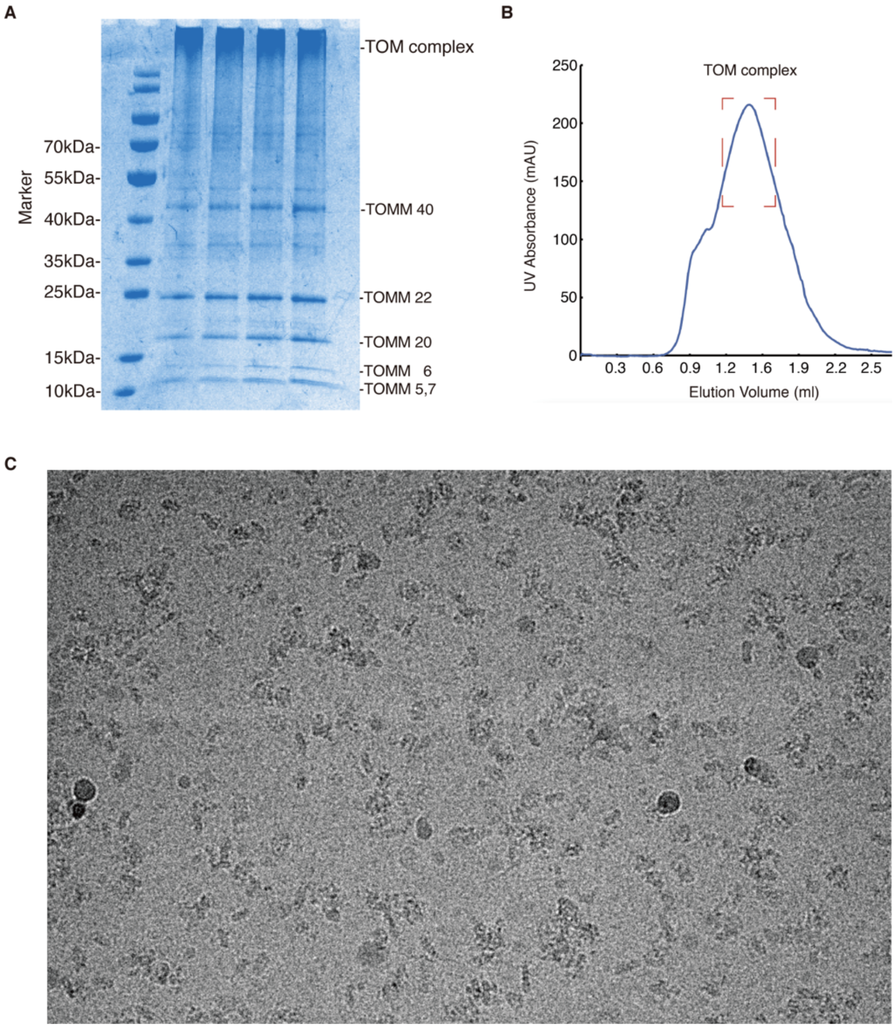

Fig. S1. Biochemical and cryo-EM analysis of the TOM complex. (A) Coomassie blue-stained SDS-PAGE of the TOM complex. **(B)** Superose 6 profile of the affinity purified human TOM complex. (**C)** A raw image of TOM complex.

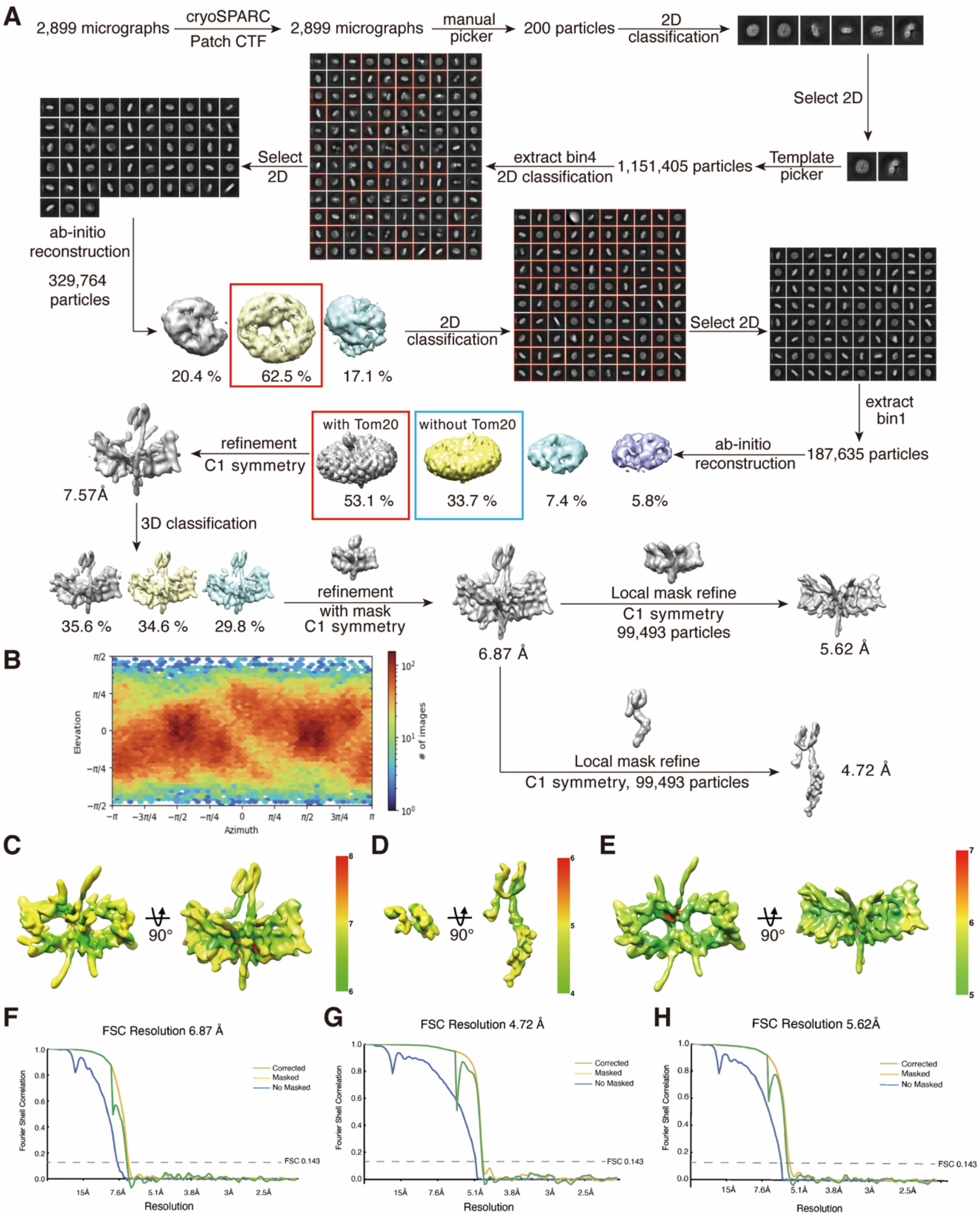

Fig. S2. Single-particle image analysis in cryoSPARC. (**A)** Flowchart of single-particle image analysis procedure in cryoSPARC. (**B)** Particle orientation distribution of the whole complex reconstruction at a resolution of 6.87 Å. **(C-E)** Local resolution maps of the whole complex **(C)**, the Tom20 **(D)** and the core region **(E)**. **(F-H)** Fourier shell correlation curves for the 3D reconstructions of the whole complex **(F)**, the Tom20 **(G)** and the core region **(H)**.

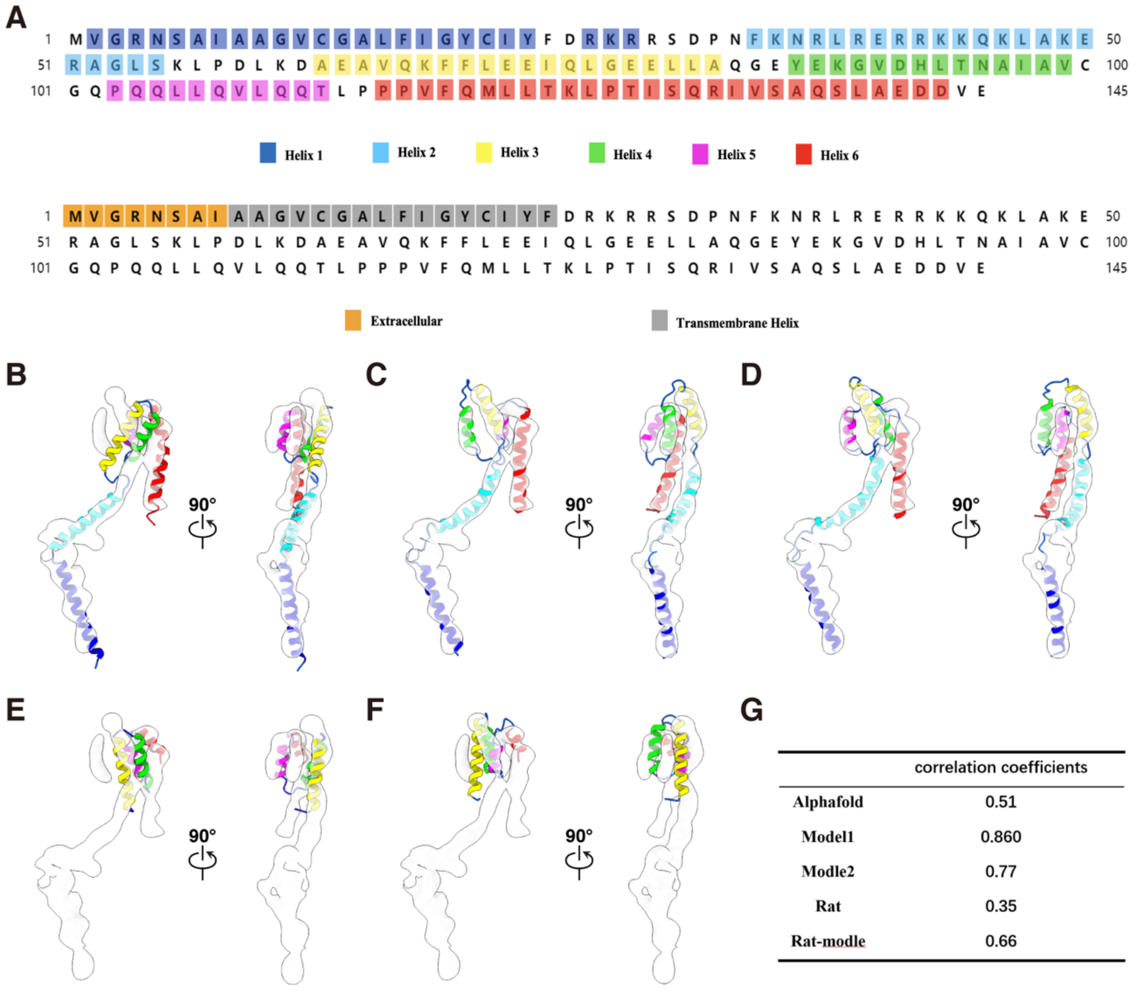

G

	correlation coefficients
Alphafold	0.51
Model1	0.860
Modle2	0.77
Rat	0.35
Rat-modle	0.66

Fig. S3. Prediction and the model fitting of Tom20.of Tom20 by PSIPRED. (A) Prediction of Tom20 by PSIPRED. **(B)** Alphafold model fits in map. **(C)** Modified Alphafold model (Model1) fits in map. **(D)** Modified Alphafold model (Model2) fits in map. **(E)** Rat model 99,493 particles fits in map. **(F)** Modified rat model fits in map. **(G)** Correlation coefficients of model fitting.

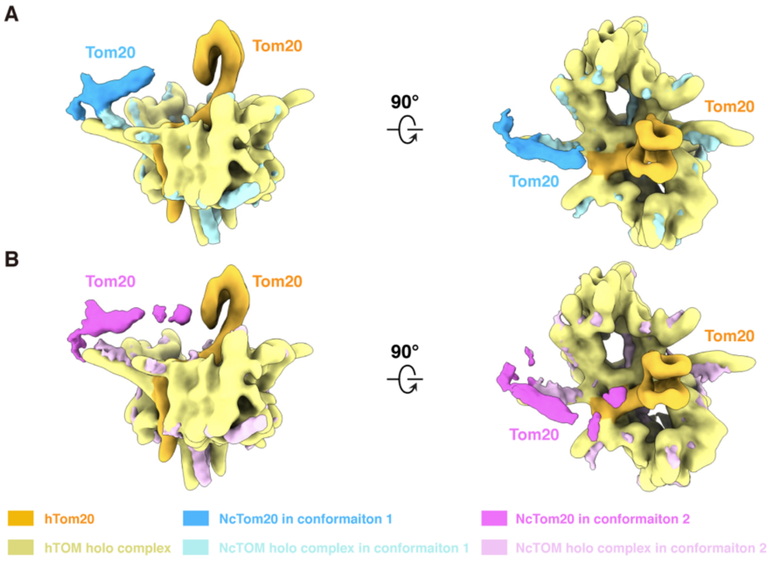

Fig. S4. Superimposition of the map of hTOM holo complex containing Tom20 with NcTOM holo complex containing Tom20. (A) Superimposition of the map of hTOM holo complex (yellow; Tom20: orange) with NcTOM holo complex in which Tom20 is in conformation 1 (cyan; Tom20: skyblue) (EMDB: EMD-15850) (40). **(B)** Superimposition of the map of hTOM holo complex (yellow; Tom20: orange) with NcTOM holo complex in which Tom20 is in conformation 2 (lightpink; Tom20: pink) (EMDB: EMD-15856) (40).

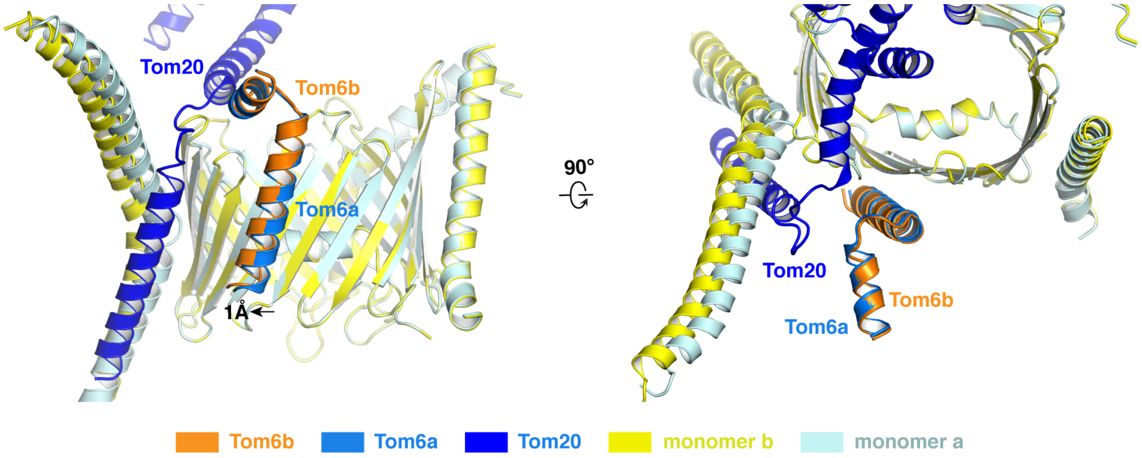

Fig. S5. Structural comparison of Tom6a and Tom6b. There is only a slight conformational change in Tom6b compared to Tom6a.

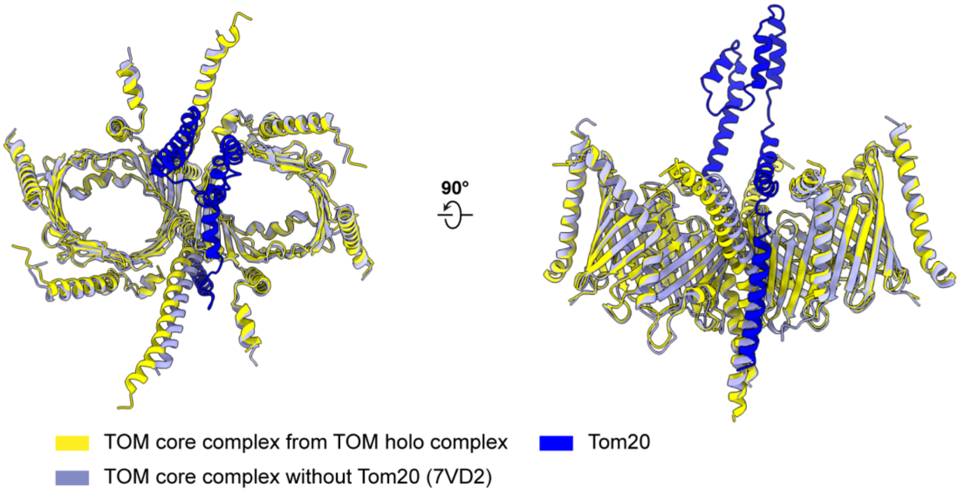

Fig. S6. Structural comparison of the TOM complex containing Tom20 and that without Tom20. Obvious conformational change exists only in the Tom22 subunit contacting with Tom20.

Table S1. Cryo-EM data collection, refinement and validation statistics.

	TOM complex PDB-8XVA EMD-38694	TOM core complex EMD-60277	Tom20 EMD-60278
**Data collection and processing**			
Magnification	81,000	81,000	81,000
Voltage (kV)	300	300	300
Electron exposure (e–/Å2)	50	50	50
Defocus range (μm)	-1.3~-1.8	-1.3~-1.8	-1.3~-1.8
Pixel size (Å)	1.0825	1.0825	1.0825
Symmetry imposed	C1	C1	C1
Initial particle images (no.)	1,151,405	1,151,405	1,151,405
Final particle images (no.)	99,493	99,493	99,493
Map resolution (Å)	6.87	5.62	4.72
FSC threshold	0.143	0.143	0.143
Map resolution range (Å)	6.0~8.0	5.0~7.0	4.0~6.0
**Refinement**			
Map sharpening B factor (Å2)	891.7	891.7	891.7
**Model composition**			
Non-hydrogen atoms	5415		
Protein residues	1100		
Ligands	0		
